# Depicted serving size: cereal packaging pictures exaggerate serving sizes and promote overserving

**DOI:** 10.1186/s12889-017-4082-5

**Published:** 2017-02-06

**Authors:** Aner Tal, Stina Niemann, Brian Wansink

**Affiliations:** 1 Cornell Food and Brand Lab, Cornell University, Ithaca, NY USA; 2 ONO Academic College, Kiryat Ono, Israel; 3 Dickinson College, Carlisle, PA USA

**Keywords:** Serving size, Consumption norms, Product packaging, Product images

## Abstract

**Background:**

Extensive work has focused on the effects of nutrition label information on consumer behavior on the one hand, and on the effects of packaging graphics on the other hand. However, little work has examined how serving suggestion depictions - graphics relating to serving size - influence the quantity consumers serve themselves. The current work examines the prevalence of exaggerated serving size depictions on product packaging (study 1) and its effects on food serving in the context of cereal (study 2).

**Methods:**

Study 1 was an observational field survey of cereal packaging. Study 2 was a mixed experimental cross-sectional design conducted at a U.S. university, with 51 student participants. Study 1 coded 158 US breakfast cereals and compared the serving sizes depicted on the front of the box with the suggested serving size stated on the nutrition facts panel. Study 2 measured the amount of cereal poured from exaggerated or accurate serving size depictions. Study 1 compared average servings via t-tests. Study 2 used a mixed model with cereal type as the repeated measure and a compound symmetry covariance matrix.

**Results:**

Study 1 demonstrated that portion size depictions on the front of 158 cereal boxes were 64.7% larger (221 vs. 134 calories) than the recommended portions on nutrition facts panels of those cereals. Study 2 showed that boxes that depicted exaggerated serving sizes led people to pour 17.8% more cereal compared to pouring from modified boxes that depicted a single-size portion of cereal matching suggested serving size. This was 42% over the suggested serving size.

**Conclusions:**

Biases in depicted serving size depicted on cereal packaging are prevalent in the marketplace. Such biases may lead to overserving, which may consequently lead to overeating. Companies should depict the recommended serving sizes, or otherwise indicate that the depicted portion represents an exaggerated serving size.

## Background

Food pictures dominate the front of packaging. Such pictures often implicitly suggest what should be an appropriate portion size. The current work focuses on the prevalence and potential effects of these implicit serving size suggestions on serving cereal.

The U.S. Food and Drug Administration (FDA) has focused regulation of serving size suggestions on the suggested serving size that are verbally stated on nutrition panels. However, serving sizes depicted on packaging may offer a more salient and powerful guide to consumption than that offered by verbally suggested serving size on nutrition labels. If depicted portion sizes are greater than suggested serving size printed on panels, they may implicitly suggest a larger consumption norm, and consequently a greater serving size. Consumers may unwittingly be primed by such suggestions to serve greater portions. Given its potential impact on consumption, such influence has implications for public policy, company practice, and consumer welfare.

Consumption norms, ideas of what quantity is appropriate for consumption, have steadily increased over the years [1]. As portion sizes have increased, so has energy intake, leading to increases in obesity rates, and concomitant health problems [2–7].

Suggested serving sizes are one means whereby consumers may determine the appropriate consumption amount [8]. However, a variety of other, potentially more powerful, elements in the food consumption environment can guide food consumption by signaling appropriate consumption amounts [9, 10]. For example, larger dishes can signal that one should consume more, and may consequently lead to increased eating [11, 12]. Such elements in the environment serve as implicit consumption norms - suggestions of appropriate consumption sizes that are implied rather than being explicitly and verbally stated (as on nutrition labels) [1].

Product packaging in particular can cue consumers to consumption norms. Traditionally, the focus of research regarding portion sizes and packaging has been on explicitly suggested serving sizes. While the correct use of nutrition panels can indeed help consumers make healthier food choices [13, 14], and determine the correct portion size in particular, the impact of labels on consumption is negligible [13, 15–17]. Many consumer segments ignore labeling information, and do not use nutrition facts panels in making their decisions [18–20].

Suggested serving size in particular may be ignored by many consumers [21]. Even when attention to suggested serving size is heightened, consumers may not apply the suggestions correctly [22]. Consumers generally have difficulty translating suggested serving sizes into action [21]. In particular, consumers may have difficulty understanding the units used in suggested portion size [23].

Visualization may play an important role in the usefulness suggested serving sizes [24]. However, consumer may have difficulty visualizing portion sizes. For example, they may not have a clear idea on how to visualize “30 g” of food such as breakfast cereal [25]. Given that food serving is visually guided, the lack of easily visualizable suggested serving sizes limit their effectiveness [21]. Visual guides may in this sense be more effective guides to consumption.

Due to their limitations, nutrition labels’ impact on changing health behavior has come into question [26]. Research findings demonstrating their lack of effectiveness has contributed to this [15, 16, 18–20]. For example, in a recent study examining the efficacy of labels in informing consumers about calories contained in products, only 54.2% of participants correctly estimated the number of calories in a Coke bottle, even after examining the label [27].

Further, verbally stated suggested serving sizes may be suspect due to company motivations. Companies may be motivated to offer serving sizes that are lower than what consumers would actually consume, because displaying lower serving sizes makes a product appear lower calorie, at least within the current labeling system in the US. This, in turn, leads to increased product choice [22].

Visual elements of product packaging, such as package size, can serve as implicit consumption norm cues and lead to increases in consumption [2, 28–30]. For example, 100-calorie packs have been shown to lead to reduced consumption [31, 32]. Similarly, just labeling a product as large or small can cue appropriate consumption amounts [33]. Visuals in general are potent at influencing consumption [28]. For example, a visual cue to appropriate portion size in the form of a visual, colored “marker” can signal the appropriate time to stop consumption [34].

The current work focuses on the influence of product imagery on packages, and specifically depicted serving sizes, on consumption. Unlike text, the images on packaging may stand out and capture consumers’ attention [35]. In general, the visuals on a package may exert a more dominant influence than words [36]. They encourage increased engagement, and can in turn play a central role in consumer decisions [37–41].

Packaging in general has been shown to have extensive influence on product judgment [42]. In the cereal domain specifically, companies have been shown to employ a variety of means to reach consumers, particularly children [43]. Packaging can generate increased product liking and increase purchases [44, 45]. Product images on packaging, in particular, provide marketers with a means of communication and persuasion [35].

The images on packaging are potent at influencing consumers, and children in particular [41, 46]. Given the extent to which children are influenced by imagery, images on food packaging have been shown to have an influence on consumption [47]. For both children and adults, the photographs and graphics on a package can provide a guide to consumption norms, how much one might believe is reasonable and appropriate to serve [8, 36]. This is particularly true given the importance of visualization to the determination of serving size [24]. If an exaggerated depicted portion suggests an exaggerated consumption norm, this may translate to increased serving, which would in turn contribute to overeating [6, 7, 12, 48, 49].

Importantly, depicted portion size may even exert an influence on children that are too young to process verbal information and so may be particularly vulnerable to visual presentation. Children may not read nutrition labels, but are able to estimate serving size based on pictures, and so may be influenced by depicted serving size [50].

Though images offer a potent channel whereby to cue appropriate consumption amounts, research on the use of such visual guides to serving sizes is notably sparse. The current studies aimed to examine whether depicted portion size may indeed affect a meaningful effect on amounts served.

Although exaggerations of depicted portion sizes occur across a wide range of products, the focus of the current paper will be on breakfast cereals, since they are frequently and widely consumed, given that their packaging clearly illustrates individual serving size (i.e., one bowl), and since they are particularly relevant for and marketed to children, which constitute a vulnerable population in formative stages of consumption habits [41, 43, 51].

The first study aimed to document deviations of depicted serving size from serving size suggested on nutrition panels. Study 2 next examined if the quantity consumers served was influenced by depicted serving size.

## Methods

The first study was exempt from IRB review due to its observational nature and lack of human subject involvement. Study 1 focused on cereals that are widely available at two national chain stores (Wal-Mart® and Target®) and a more traditional grocery store (Wegmans®) in the Northeast during the summer of 2014. The only cereals excluded were ones that did not depict the cereal in a bowl or in a way which allowed fair estimation of quantity. The sample of 158 cereals included national and store brand cereals marketed to both adult and children.

The basic procedure for estimating quantity involved using the number of pieces depicted to determine the size of the depicted bowl. First, the diameter of each cereal piece in actuality (vs. the depiction) was measured. Then, an estimate of the size of the depicted bowl was calculated by counting the number of depicted pieces that fit across the bowl. For instance, if each piece of cereal was .5 in. in diameter, and 11 pieces fit across the depicted bowl, bowl diameter was calculated as .5 × 11 = 5.5 in.. The reliance on objective cereal diameter allowed us to correct for the fact that some packages depict a magnification of the real product. For cereals that are not consistently the same size, such as frosted flakes, random pieces were chosen.

Once the correct diameter of the depicted bowl for each cereal was determined, the closest matching bowl was chosen from a wide variety of bowls purchased for the study. Using this matching bowl, the researchers poured half a cup of milk (4 oz) into the bowl (as per the suggested serving size of milk for all 158 cereals), and added cereal from a standardized 100 g graduated laboratory cylinder. Researchers poured cereal until—visually-- the amount in the bowl matched the depicted amount on the cereal box. Researchers then weighed the amount of cereal remaining in the tube and subtracted that amount from 100 g to determine the amount in the bowl. That amount was also used to calculate the calories in the cereal served.

These measurement procedures for determining the amount of cereal in depicted bowls were repeated using a second coder for inter-rater reliability, and repeated for one of the coders for intra-rater reliability. This was done with a 20% subset of cereals (*n* = 30 representing the full range of volume levels). The analysis revealed high inter-rater reliability (.89) and high intra-rater reliability (.92), indicating a high though not perfect match.

The IRB approved second study was a mixed, within-subject and between-subject design which varied the two serving sizes (exaggerated, multiple serving size versus the recommended single-serving size) between the first cereal participants saw and the second. In all cases the first cereal given was Frosted Flakes®, and the second was Lucky Charms®. Note that some participants saw the multiple-serving size first and the single-serving size second, and some vice versa. However, in all cases participants viewed Frosted Flakes first. The cereals were selected as representative of the general presweetened cereal category because they are popular, have a relatively large market share each, are widely available, and are sufficiently different from each other to contribute to the generalizability of our findings. Note that suggested serving sizes for both cereals varies on commercial packages, but revolves around 30 g.

Participants for Study 2 were 51 students (69% female, mean age 22.3 range 18–55) at a large Northeastern American university. The study was run in the fall semester of 2014. Participants were recruited in an introductory, mostly undergraduate Consumer Behavior class offered as part of the school of Applied Economics. The students were recruited at the class meeting during the morning hours and were asked to participate in the study taking place during class time in exchange for extra credit. All participants asked agreed to participate in the study, and there were no missing data from those participating. Participants provided informed consent, and were then randomly divided into four small groups of 10–15. The study was run between 8:45 and 9:30 AM.

Each group was told they were going to be asked a number of questions related to their daily routine. Individual participants from each group were led into a room where a standard commercially available cereal box was available. Each participant completed the study on their own, without observing other participants.

Participants were asked to pour the amount of cereal they would typically pour themselves if they were going to have cereal. Importantly, no comment was made about the appropriate serving sizes or the cereal boxes. Participants could observe the boxes freely and examine nutrition information or ignore it as they wished. Participants served themselves cereal but were not required to consume it.

Participants were randomly assigned to experimental rooms, with each participant going to one room. In two of the experimental rooms, participants poured from cereal boxes of Frosted Flakes that depicted an exaggerated, multiple serving size box – the standard, commercially available version of the product. In the other two rooms, the other half of the participants were given the same size boxes of cereal, with the depicted amount of cereal on the label modified by a graphic artist to match the single-serving suggested on the side panel of the box. Images were manipulated using Photoshop™ CC (Adobe 2013).

After participants served themselves, bowls were collected and their contents were surreptitiously weighed and recorded in a staging room near the kitchen. During this time, participants completed an unrelated 20-min survey.

Afterwards, they were invited to a different room where the pouring procedure was repeated with a second cereal, Lucky Charms. Participants who poured from a single-serve depiction box of Frosted Flakes before now poured from a multiple-serving box of Lucky Charms, and vice versa.

Before leaving the study, participants reported their gender, age, height and weight. In serving studies where participants serve but do not eat the food, it is conventional to eliminate observations that deviate beyond either two or three standard deviations from the mean. One observation was eliminated where a participant served 253 g of cereal, three SDs above the mean (SD = 50.79), potentially due to accidental pouring.

Since calories per 100 g were nearly identical for both cereals, with Frosted Flakes at 378 and Lucky Charms at 380, average calories per cereal were calculated using the average of 379 calories per 100 grams.

A mixed model with cereal type as the repeated measure was used for analysis [52, 53]. The model included for depicted size and cereal type and their interaction. The covariance structure was specified as compound symmetry. A compound symmetry structure assumes variances and covariances are homogeneous for the repeated observation [54, 55]. Though its use has originated in animal studies, it has become widespread across disciplines, including medicine [56]. Essentially, use of compound symmetry requires an assumption that variance will not radically vary between repeated measures – in our case, cereal types. Since we do not have grounds to assume the cereals behave differently in this case, and similar variances for both cereals are expected and can be observed in examining data distribution, the assumption is appropriate. However, note that results hold with an unstructured covariance matrix, making no assumptions about the structure of the variance.

## Results

The first study revealed that the serving sizes depicted on the front of 158 cereals were an average of 64.7% greater than the serving sizes stated on nutrition panels of those cereals. The average depicted serving size was 220.57 calories (SD = 117.03), whereas average suggested serving size was 133.85 calories (SD = 39.03). This difference of 86.72 calories was significant at the *p* < .001 level [t (158) = −10.03]. Average depicted serving size in grams was 58.9 g (SD = 30.33), while average suggested serving size on the nutrition panel was 35.64 g (SD = 10.91), a difference of 65.26%. Table 1 below presents calorie differences between suggested and depicted serving sizes. Differences in percentages between calories and grams emerged due to different calorie densities.

**Table 1:** Average suggested vs. Depicted serving size cereal calories

*N* = 158	Mean calories (SD)
Suggested Serving calories	133.85 (39.03)
Depicted Serving calories	220.57 (117.03)
Difference in calories	86.74 (108.33)
Percent Difference in Calories	71.27%
Paired Sample t-test (*p*-value)	−10.03*** (.000)

*** < 0.001 for *t*-test values

Across a wide range of cereals, depicted portion sizes are markedly higher than those suggested on the nutrition panels of those same cereals. The second study was conducted to determine if consumers were influenced by such depictions of serving size to serve themselves greater amounts of cereal.

Participants poured 17.8% more cereal (162 vs. 137 calories) when pouring from cereal boxes which depicted multiple servings, vs. when pouring from cereal boxes modified to depict only one serving. That is, serving was increased when they served from a package that depicted a multi-serving portion than when it depicted a modified single-portion (see Table 2). Adjusted mean serving size was 42.62 g (SD = 21.08) for the commercial (multi-serving) package, and 36.17 g (SD = 28.44) for the modified (single-serving) package. Depicted serving size had a significant main effect on the amount of cereal served [F(1, 46) = 5.78, *p* = .02]. Effect size, as measured by Cohen’s d, was .69. The interaction between cereal type and depicted size was not significant: F(1, 46) = .46, *p* = .46.

**Table 2:** Influence of depicted portion size on amount of cereal served, in Study 2

	N	Cereal Grams Served Mean(SD)	Calories Poured Mean(SD)
Single serving (modified package)	50	36.04 (21.08)	136.97 (80.1)
Multiple servings (commercial package)	50	43 (28.44)	163.4 (108.06)
F(1, 46)		5.78	5.78
p		0.02	0.02

Serving from the commercial (multi-serving) package was 42% over suggested serving size of 30 g, as opposed to an already exaggerated 20% over suggested serving size with our modified, correctly depicted serving size. Results are summarized in Table 2. Such increase in food consumption overall would translate, over time, to weight gain to a potentially harmful degree.

Including a compilation of potentially influential variables: Age, Gender, and BMI in the model along with their interactions with depicted size retained the significant effects of depicted serving size: F(1, 46) = 5.76, *p* = .02. Effect size calculated with Cohen’s d was unchanged at .69. Including interactions of size with each of these variables revealed no significant interaction effects for any of the factors. In other words, the effects of depicted serving size on self-serving of cereal as robust across these variables.

## Discussion

Study 1 demonstrated that depicted serving size on the front of cereal packaging is 65.26% larger than suggested on nutrition facts panels. Study 2 demonstrated that depicted portions adjusted to match suggested serving size led to reduced serving amounts compared to standard depictions on commercial packages. Participants serving from commercial packages 18% more cereal relative to those seeing depictions matching suggested serving sizes, 42% over the serving size suggested on the nutrition panel.

Consumption norms have an enormous impact on how much consumers eat. Consumption norms can take the form of package sizes, a friend’s serving size, and even plate, bowl, and serving utensil size. All of those can cue consumers to larger servings, and consequently, increased consumption [9–11, 31]. Suggested portion sizes can serve as one cue for appropriate consumption levels [8]. The current work demonstrated that *depictions* of portion size can serve as an implicit consumption norm, which may subsequently lead to increased consumption.

Though the current study focused on amount served rather than actual consumption, participants were asked to serve the amount they would take for consumption. In addition, past research demonstrates a clear relation between portion served and amount eaten, such that larger portions result in increased eating [6, 7, 57, 58]. Hence, increased consumption norms depicted on packaging may well lead to overeating [12, 59].

Consumption norms may be altered by other factors related to depicted portions, such as the amount of food depicted in advertising. For instance, if beer ads repeatedly depict beer drinkers with a six-pack instead of a single beer, this can alter perceptions of how many beers it is normal to consume at one time. Similarly, depicting multiple units on a package (or in an ad) could lead consumers to consume more. For example, seeing three versus two ice cream scoops, or three wrapped snacks vs. one on packaging, may suggest those are normal amounts to eat, and consequently lead to overeating.

There are several limitations of this research that will hopefully open a rich new area of future research on consumption norms. The current studies only examined the disparity between depicted and suggested serving size for one product category – American breakfast cereals. It would be important to know how wide-spread the influence of depicted serving sizes is across countries and product categories. While the focus here was on one food product category, similar findings should exist for any product that comes in a multi-serving package and that can have variable serving quantities.

One limitation of the first study involved the limited geography of the sample. The 158 national brands and store brands that were examined were from three stores in the same vicinity. While it is true that stores in other regions or countries may have other cereals bearing potentially different pictures, it is important to note that there was very little observed difference among cereals – most had an estimated depicted serving size deviation that was at least 50% larger than the single-size serving on the side panel. Moreover, many of these national brands have a significant if not dominant international market share. A further limitation of the first study is that it relied on an estimation procedure that by necessity included some assumptions. However, given the magnitude of the effects found, small deviations from actual content of depicted cereal bowls would not substantially alter the findings.

Study 2 examined whether depicted portion size deviations influenced how much cereal a homogeneous group of people served themselves on a single occasion in a fairly realistic consumption environment. There are at least four limitations to this study: it examined serving behavior (versus consumption behavior), it used a homogeneous student sample (versus a heterogeneous one) that may not be representative of the general population, it measured single-occasion serving (versus multiple occasion serving), and it used a lab environment designed to mirror a realistic consumption environment (versus a randomized control trial).

This study can serve as the basis for a randomized control trial (RCT) of a large heterogeneous population whose cereal consumption behavior is studied over time (multiple occasions), in actual home environments. Such a study could further substantiate our findings, and help generalize them to ecologically valid conditions.

### Implications for research and practice

Recent FDA efforts have focused on making portion sizes more transparent. To date, regulations have focused on whether calories listed on packages display calories for the entire package or for a single serving [8]. Many manufacturers have lobbied to highlight traditionally modest single-serving sizes on their package labels. This allows them to display low calories – which is good for attracting calorie conscious shoppers.

Examining serving behavior is a critical first step in eventually examining actual consumption behavior, which may have important implications for public health. Since aggregated studies have suggested that adults may consume close to 92% of what they serve themselves, overserving would lead to overeating, and may so be a meaningful factor contributing to obesity [7, 60].

If additional research further substantiates our findings on the influence of depicted serving sizes, responsible cereal manufacturers may wish to adjust depicted serving sizes to better match recommended single-serving portion sizes.

From a policy perspective, having companies self-monitor depicted serving size deviations on their packaging could save valuable overhead costs required for external monitoring. Furthermore, it would still allow companies more flexibility in how they depict portion size on a package and suggest the correct serving size, for instance through graphics, icons, or what is said on the package (“family-sized,” “share with a friend,” “lasts a week”). Companies may also solve the issue by including a scoop within packages that matches suggested serving size.

From a consumer perspective, the current research underscores that portion sizes depicted on packaging might lead to overserving. One solution might be to advise consumers to focus on recommended serving size, rather than front-of-package depictions. However, this requires daily vigilance and an uncommon ability to envision what a single 30 g serving would look like.

One viable solution is to simply transfer cereal out of its packaging and into resealable serving containers. Once stored in a single-serve container, cereal packaging no longer provides depicted serving norms and the packaging is no longer there to provide visual temptation. In addition, using pre-measured single-serve packages can allow pre-portioning of cereal to control portion sizes.

## Conclusions

Cereal packages display serving sizes approximately 65% larger than suggested serving sizes. The serving size depicted on packaging in turn influences the amount of cereal consumers serve themselves, such that larger depicted serving sizes lead to larger servings, regardless of the verbally suggested serving size on nutrition labels. The prevalent exaggerated portions depicted on packages of cereals as well as other food products may contribute to overeating and obesity, and consequently constitute a factor with a potentially adverse impact on public health. Companies, consumers and regulators alike should consider these effects in determining appropriate packaging displays and serving amounts.
