# Erratum to: Depicted serving size: cereal packaging pictures exaggerate serving sizes and promote overserving

**DOI:** 10.1186/s12889-017-4185-z

**Published:** 2017-03-17

**Authors:** Aner Tal, Stina Niemann, Brian Wansink

**Affiliations:** 1 Cornell Food and Brand Lab, Cornell University, Ithaca, NY USA; 2 ONO Academic College, Kiryat Ono, Israel; 3 Dickinson College, Carlisle, PA USA

## Erratum

The authors re-ran the analyses for both studies, hoping to correct any minor discrepancies prior to publication of this article [1]. They regret having missed the last chance to make minor adjustments to the article prior to publication. Due to this, they include corrections to the stats as described below. In summary, corrections to Study 1 include minor tweaks to statistics of no more than 1 calorie, adjusting percent difference in calories by about 1%. Corrections to Study 2 include analysis dropping both observations from one participant who overserved by more than 3 SDs, as described below, and minor adjustments to the statistics. No findings or conclusions are impacted by these corrections. In fact, by and large the adjustments offer slightly stronger results. Corrected tables include the updated numbers, easily comparable to the manuscript initially published online.

**Abstract**

Corrections to numbers reported in results, as follows: Results: Study 1 demonstrated that portion size depictions on the front of 158 cereal boxes were 65.84% larger (221 vs. 133 calories) than the recommended portions on nutrition facts panels of those cereals. Study 2 showed that boxes that depicted exaggerated serving sizes led people to pour 20% more cereal compared to pouring from modified boxes that depicted a single-size portion of cereal matching suggested serving size. This was 45% over the suggested serving size.

**Methods**

Study 2. Observations from one participant, who served 253 grams of cereal, three SDs above the mean (SD = 50.79), were eliminated from analysis.

**Results**

Study 1. The first study revealed that the serving sizes depicted on the front of 158 cereals were an average of 65.84% greater than the serving sizes stated on nutrition panels of those cereals. The average depicted serving size was 220.57 calories (SD = 117.03), whereas average suggested serving size was 133 calories (SD = 40.34). This difference of 87.57 calories was significant at the *p* < .0001 level [t(157) = -10.15]. Average depicted serving size in grams was 58.9 grams (SD = 30.33), while average suggested serving size on the nutrition panel was 35.64 grams (SD = 10.91), a difference of 65.27%.

Study 2. Participants poured 20% more cereal (164 vs. 137 calories) when pouring from cereal boxes which depicted multiple servings, vs. when pouring from cereal boxes modified to depict only one serving. That is, serving was increased when they served from a package that depicted a multi-serving portion than when it depicted a modified single-portion (see Table 2). Mean serving size was 43.37 grams (SD = 28.96) for the commercial (multi-serving) package, and 36.04 grams (SD = 21.08) for the modified (single-serving) package. Depicted serving size had a significant main effect on the amount of cereal served [F(1, 46) = 5.76, *p* = .02]. Effect size, as measured by Cohen’s d, was .69. The interaction between cereal type and depicted size was not significant: F(1, 46) = .46, *p* = .5.

Serving from the commercial (multi-serving) package was 45% over suggested serving size of 30 grams, as opposed to an already exaggerated 20% over suggested serving size with our modified, correctly depicted serving size.

**Discussion**

Study 1 demonstrated that depicted serving size on the front of cereal packaging is 65.27% larger than suggested on nutrition facts panels. Study 2 demonstrated that depicted portions adjusted to match suggested serving size led to reduced serving amounts compared to standard depictions on commercial packages. Participants serving from commercial packages served 20% more cereal relative to those seeing depictions matching suggested serving sizes, 45% over the serving size suggested on the nutrition panel.

Tables 1 and 2 are also corrected. These are presented below:

**Table 1:** Average suggested vs. depicted serving size cereal calories (Cereal only)

*N* = 158	Mean calories (SD)
Suggested Serving calories	133 (40.34)
Depicted Serving calories	220.57 (117.03)
Difference in calories	87.57
Percent Difference in Calories	65.84
*Paired Sample t-test (p-value)*	-10.15 (<.0001)

**Table 2:** Influence of depicted portion size on amount of cereal served, in Study 2

	N	Cereal grams served Mean (SD)	Calories poured Mean (SD)
Single serving (modified package)	49	36.04 (21.08)	136.61 (79.89)
Multiple servings (commercial package)	49	43.37 (28.96)	164.36 (109.77)
F(1, 46)		5.76	5.76
P		0.02	0.02
